# The burden of acute respiratory infections in crisis-affected populations: a systematic review

**DOI:** 10.1186/1752-1505-4-3

**Published:** 2010-02-11

**Authors:** Anna Bellos, Kim Mulholland, Katherine L O'Brien, Shamim A Qazi, Michelle Gayer, Francesco Checchi

**Affiliations:** 1 Disease Control in Humanitarian Emergencies, World Health Organization, Geneva, Switzerland; 2 Department of Epidemiology and Population Health, London School of Hygiene and Tropical Medicine, London, UK; 3 Department of International Health, Johns Hopkins University Bloomberg School of Public Health, Baltimore, Maryland, USA; 4 Newborn and Child Health and Development, World Health Organization, Geneva, Switzerland; 5 Department of Infectious and Tropical Diseases, London School of Hygiene and Tropical Medicine, London, UK

## Abstract

Crises due to armed conflict, forced displacement and natural disasters result in excess morbidity and mortality due to infectious diseases. Historically, acute respiratory infections (ARIs) have received relatively little attention in the humanitarian sector. We performed a systematic review to generate evidence on the burden of ARI in crises, and inform prioritisation of relief interventions. We identified 36 studies published since 1980 reporting data on the burden (incidence, prevalence, proportional morbidity or mortality, case-fatality, attributable mortality rate) of ARI, as defined by the International Classification of Diseases, version 10 and as diagnosed by a clinician, in populations who at the time of the study were affected by natural disasters, armed conflict, forced displacement, and nutritional emergencies. We described studies and stratified data by age group, but did not do pooled analyses due to heterogeneity in case definitions. The published evidence, mainly from refugee camps and surveillance or patient record review studies, suggests very high excess morbidity and mortality (20-35% proportional mortality) and case-fatality (up to 30-35%) due to ARI. However, ARI disease burden comparisons with non-crisis settings are difficult because of non-comparability of data. Better epidemiological studies with clearer case definitions are needed to provide the evidence base for priority setting and programme impact assessments. Humanitarian agencies should include ARI prevention and control among infants, children and adults as priority activities in crises. Improved data collection, case management and vaccine strategies will help to reduce disease burden.

## Introduction

### Infectious diseases in crisis-affected populations

Health crises may be defined as the occurrence of morbidity and mortality in excess of secular trends, due to natural or man-made disasters [1]. With the exception of natural disasters and some recent wars (e.g. Iraq, Lebanon), the excess death toll in crises appears to be mainly "indirect". Excess deaths are due to an increased risk of disease and case-fatality brought about by conditions such as displacement into overcrowded camps, food insecurity, and breakdown of public health services, rather than the direct effects of the crisis [1,2].

While most indirect excess mortality during crises is of infectious aetiology, data on the relative contribution of various infectious diseases are scarce. Diseases that cause a visible impact through dramatic epidemics, such as measles, cholera, dysentery and malaria [3], are usually considered the top threats during humanitarian relief operations. Accordingly, humanitarian agencies have emphasized mass measles vaccination, improved water and sanitation, and distribution of insecticide-treated materials as priority preventive interventions during the acute emergency phase [4-7].

By contrast, acute respiratory infections (ARI) have received far less attention in humanitarian relief policies and programmes, despite being the largest baseline contributor to disability-adjusted life-years (DALYs) lost and the leading single cause of mortality among children under 5 y worldwide [8-11].

### Epidemiology of acute respiratory infections

ARIs may be classified into upper (AURI) and lower (ALRI) acute respiratory infections, depending on the main organs affected (nose, sinuses, middle ear, larynx and pharynx versus trachea, bronchi and lungs). AURIs are generally mild in nature and most often caused by viruses, sometimes with a bacterial component as in some cases of sinusitis and otitis media[12]. The overwhelming majority of ARI deaths and severe illness episodes are due to ALRIs, consisting mainly of pneumonia [13]. Nearly all severe ALRI episodes occur in children under 5 y, the elderly and immunocompromised individuals (e.g. HIV-infected). Globally, about 4.2 million ALRI deaths are estimated to occur among all age groups; of these 1.8 million are estimated to occur among children 1-59 m [14].

The aetiology of ALRI, and pneumonia in particular, is difficult to establish [15,16]. Collecting body fluid specimens for microbiologic diagnosis from the site of infection can only be done in a small proportion of cases. Aetiology studies are therefore based on either insensitive or non-specific indirect methods such as blood culture, serology and microbiologic assessments of the upper airway. Based on these studies, about one fourth to one half of childhood pneumonia cases appear to have a primarily viral aetiologic agent, including human respiratory syncytial virus (RSV), parainfluenza and influenza viruses [12,17]. Half or more are due to bacteria, with some presenting as a secondary infection of an acute viral process, including measles, influenza, or RSV [13]. Pertussis may also predispose to bacterial superinfections. HIV infection increases the risk of both *Streptococcus pneumoniae *and *Haemophilus influenzae *type b, together responsible for about half of pneumonia deaths, by 7-40 fold [18,19]. Though evidence is incomplete, bacterial episodes of pneumonia are believed to feature a higher severity and case-fatality ratio (CFR) than viral episodes [12], hence the emphasis on antibiotic treatment of children with pneumonia. Among children under 2 y, *S. pneumoniae *and *H. influenzae *type b (Hib) are estimated to cause 36% and 22% of radiological pneumonia cases respectively [18,19], but other pathogens, including *Staphylococcus aureus*, *Mycobacterium tuberculosis *and non-typeable *H. influenzae*, likely play a substantial though poorly understood role [12,13,17]. The 2009 H1N1 influenza pandemic may alter the above patterns substantially, although historical and more contemporary evidence points to the importance of *S. pneumoniae *as a risk factor in fatal influenza cases [20].

### Rationale for this review

In any pre-crisis setting, ALRI (and thus ARI as a whole) will usually be the leading infectious cause of mortality, and among the top three causes of death overall. Reasoning suggests that the onset of a crisis should result in an even higher ALRI disease burden, both in absolute and relative terms. Risk factors that would specifically manifest themselves during crises include (i) malnutrition (both chronic and acute); (ii) inadequate shelter conditions, mainly due to displacement or destruction of houses, and resulting in exposure to cold temperatures and/or to indoor air pollution from use of solid fuels; (iii) overcrowding due to displacement into camps or into host households (the latter resulting in overcrowded living and sleeping quarters); (iv) decreased coverage of Expanded Programme of Immunization (EPI) interventions, including measles, pertussis and (where already implemented) Hib vaccination, particularly in settings affected by chronic insecurity; and (v) lack of or delay in diagnosis and treatment due to insecurity and breakdown in health services [1,21]. Other factors, including psychological stress, exhaustion, increased frequency of low birth weight and prematurity, exposure to toxic weapons and airborne particulates in the aftermath of volcanic eruptions may also play a role.

These factors could affect the risks of transmission and infection, progression to disease among those infected, progression to severe disease (e.g. pneumonia) among those ill, and/or case-fatality. Furthermore, some of these factors increase risk in a synergistic manner, and their combination could result in bona fide epidemics of ARI pathogens, defying expectations of ARI as a high-incidence but endemic condition. Displacement increases the proportion of the population that needs to be immune in order to maintain herd immunity. This in turn facilitates epidemics of measles that lead to a high burden of secondary pneumonia. Measles can also exacerbate nutritional emergencies, and the resulting malnutrition further increases susceptibility to pneumonia. Though crises do not necessarily result in higher HIV prevalence, a high pre-crisis HIV prevalence would likely interact with nearly all other risk factors for ALRI, and the faster progression of AIDS cases would result in a greater burden of opportunistic respiratory disease.

Unlike for other high-burden infectious diseases, there are no specific recommendations for prevention and control of ARI in crises. To strengthen the evidence base on which such recommendations could be formulated, we conducted a systematic review of the burden of ARI in crisis-affected populations.

## Methods

### Eligibility criteria

We included studies in this review if they presented quantitative data on (i) ARI, upper or lower respiratory tract infections (AURI, ALRI), pneumonia or any other disease classifiable as ARI under the International Classification of Diseases, version 10 (codes J00-J22) [22]: this includes influenza but not measles or tuberculosis; we excluded reports of ARI as a nosocomial infection; (ii) populations who at the time of the study were affected by natural disasters, armed conflict, forced displacement, and nutritional emergencies (we excluded reports of refugees resettled in third countries and military populations); and (iii) any indicator of burden, namely incidence or prevalence of infection or disease, proportional morbidity, attributable mortality rate, proportional mortality, or CFR.

We excluded reports in which infectious and non-infectious (e.g. asthma) respiratory diseases were grouped together, the study design (e.g. review of patient records) or the setting (e.g. outpatient) were unclear, and/or the diagnosis was not based on examination by a clinician or a structured verbal autopsy questionnaire.

### Information sources and search strategy

Prior to performing the literature review, we searched the Database of Abstracts and Reviews of Effects (DARE) and the Cochrane Database of Systematic Reviews for reviews of ARI in crisis-affected populations, but found none.

We then identified relevant peer-reviewed articles published between January 1980 and June 2009 in English, French, Spanish, Portuguese, Italian and German, and presenting data on ARI due to any aetiology within populations affected by natural disasters, displacement, armed conflict or nutritional emergencies. To do this, we searched the EMBASE, CAB Abstracts and Medline databases (both the PubMed and OvidSP interfaces were used for the latter), according to the following five steps.

First, we did a medical subject heading (MeSH) thesaurus search, combined with text word searches to obtain a full set of synonyms for the various combinations of three general concepts: [type of ARI disease] & [disease burden indicator] & [type of crisis]. Searches including names of specific pathogens were also done, but did not yield additional reports. For details of keywords used for each concept, and the number of abstracts generated by this search strategy, see Additional File 1, Box 1.

Second, we did a search by specific armed conflict (see Additional File 1, Box 2): [country] & [war] & [respiratory OR disease]. We used the UCDP/PRIO Armed Conflict Dataset, version 4-2009 [23]http://www.prio.no/CSCW/Datasets/Armed-Conflict/UCDP-PRIO/ to select 37 countries (Additional File 1, Box 3) for this search (namely, those in which ≥ 1000 or more battle related deaths were reported during any year since 1980).

Third, so as to capture ARI reports among refugees fleeing to neighbouring countries, we did a simple keyword search using the terms "respiratory & refugee".

Fourth, we did a search by type of natural disaster (earthquake, tsunami, flood, famine, drought, volcano): [disaster type] & [respiratory or ALRI] & [health or illness or infection or disease] (Additional File 1, Box 4).

Fifth, we scanned references of key articles, including reviews and meta-analyses, for relevant related publications.

Finally, we also searched the websites of the Hib Initiative http://www.hibaction.org, the Pneumococcal Accelerated Development and Implementation Plan (PneumoADIP; http://www.preventpneumo.org) and the World Health Organization http://www.who.int for relevant sources, including grey literature.

### Data extraction

One of us (AB) extracted onto an Excel template key variables for each study (years data collection started and ended; region and type of population, e.g. refugee camp; setting of the study, e.g. outpatient clinics; type of study design, e.g. prospective surveillance, household survey; ARI case definition provided; a brief description of the main data quality and selection biases as noted by the reviewer), and for each indicator reported (number of cases, denominator at risk, frequency and duration of follow-up for prospective surveillance studies; rank of ARI among other conditions for which the same indicator was also reported), with as much age stratification as was provided. One of us (FC) checked entries for accuracy. We did not contact authors to obtain further data.

### Statistical analysis

As only three of the studies included in the review provided clear case definitions for ARI (see below), we felt that studies were not sufficiently comparable to perform pooled analyses. Instead, we present summary data for each study in descriptive tables and provide ranges for the key burden indicators. Whenever studies provided data for crisis settings and a comparable pre-crisis period or control setting, we used these data to calculate crisis versus non-crisis relative risks. When no direct comparisons for non-crisis settings were available, we used major studies of the global ARI burden as the baseline. Lastly, where the burden indicators were not reported but enough data (i.e. numerator, denominator at risk) were provided to compute them, we did so ourselves.

## Results

### Study selection

We screened 5097 abstracts, out of which we retrieved and assessed for eligibility 99 reports, of which 63 did not meet inclusion criteria for one or more of the following reasons: the population was not crisis-affected as defined by the criteria (7 reports), no quantitative data on ARI burden were reported (22), data reported were insufficient to construct burden indicators (5), data were for a mixed group of crisis and non-crisis affected people (1), no ARI case definition was provided (9), the case definition potentially included chronic respiratory diseases (10), the diagnosis was made retrospectively based on reported symptoms (14), the diagnosis was not made by clinicians (12), the health care setting in which data were collected was unclear (3), and the data were also presented in another report reviewed (2).

### Study characteristics

Thirty-six studies were included in the review. Four were published in the 1980s, 15 in the 1990s and 17 since 2000. Three reports were from countries in the WHO Region of the Americas, 10 from the African Region, nine from the Eastern Mediterranean Region (which includes Somalia, Sudan, Pakistan and Afghanistan), three from the European Region, eight from the South-East Asia Region, and three from the Western Pacific Region http://www.who.int/about/regions/en/index.html.

Twenty-five studies (69.4%) reported on populations affected by armed conflict. Most of these (n = 18) were refugee populations, of which 11 occurred during the acute emergency phase (Cambodians in Thailand, 1980 [24]; Somalis in Ethiopia, 1989 [25]; Iraqi Kurds in Iran, 1991 [26]; Bhutanese in Nepal, 1992 [n = 2] [27,28]; Burundians in Rwanda, 1993 [29]; Rwandans in Zaire, 1994 [n = 2] [30,31]; Kosovars in Albania, 1999 [n = 3] [32-34]), and seven during the post-emergency phase (Guatemalans in Mexico, 1983 [35]; Nicaraguans in Costa Rica, 1986 [36]; Afghans in Pakistan, 1986 [37]; Cambodians in Thailand, 1987-1991 [n = 2] [38,39]; Vietnamese in Hong Kong, 1991-1992 [40]; Sudanese in Uganda, 1992-1994 [41]). All of the above refugee populations were living in camps with the exception of some Kosovar refugees in Albania.

Only three studies reported on internally displaced people (IDPs): northern Ugandans (1992-2002) [n = 2] [42,43] and IDPs in Darfur, Sudan (2004) [44], both mainly living in camps. Four studies reported on urban, non-displaced populations living in armed conflict areas, of which one covered the acute emergency period (Bissau, Guinea Bissau, 1998-1999) [45] and three the post-emergency or early recovery period (Kabul [46] and Herat [47], Afghanistan, 2002-2003; Monrovia, Liberia, 2005 [48]).

Finally, 11 studies (30.6%) described populations in the immediate aftermath of natural disasters, including earthquakes in Japan (1995) [49], Taiwan (1999) [50], Iran (2003) [n = 2] [51,52] and Pakistan (2005) [n = 2] [53,54]; floods in Bangladesh (1988) [55], Mozambique (2000) [56] and India (2001) [57]; tsunami waves in Indonesia (2004-2005) [58]; and a volcano eruption in Nicaragua (1992) [59].

### ARI-attributable morbidity

#### Incidence

We found seven reports of the incidence rate of ARI (Table 1). Only one study [26], however, measured community incidence, finding a ratio of about 5-12 AURI cases to 1 ALRI case: because no age stratification was reported, this study is not easily comparable to non-crisis settings, for which incidence rates among children under 5 years are well described. In three other studies, ALRI incidence rates were in the range of 0.6-1.4 per 1000 person-weeks: however, these reflected only cases presenting for treatment at clinics.

**Table 1:** Reports of ARI incidence rate in crisis-affected populations, by setting (community, outpatient, inpatient).

**Disease**	**Ref**.	**Population (year)**	**Study design**	**Case definition, as reported**	**Incidence rate as cases per 1000 person-weeks (rank if reported)**	
					**By age group**	**All ages**
		
**Community**						
AURI	[26]	Iraqi-Kurdish refugees in Iran (Noswood border camp) (1991)	Enhanced community surveillance	Upper respiratory tract infection		11.2
	[26]	Iraqi-Kurdish refugees in Iran (Sarayas border camp) (1991)	Enhanced community surveillance	Upper respiratory tract infection		24.9
ALRI	[26]	Iraqi-Kurdish refugees in Iran (Noswood border camp) (1991)	Enhanced community surveillance	Lower respiratory tract infection		2.1
	[26]	Iraqi-Kurdish refugees in Iran (Sarayas border camp) (1991)	Enhanced community surveillance	Lower respiratory tract infection		2.0
		
**Outpatient**						
ARI	[59]	Residents of Malpaisillo, Nicaragua after volcano eruption (1992)	Clinic-based surveillance	Acute respiratory illness	<12 m: 75.0; 12-59 m: 37.2; 5-14 y: 18.3; 15-49 y: 12.2; ≥ 50 y: 10.8	
	[59]	Residents of Telica, Nicaragua after volcano eruption (1992)	Clinic-based surveillance	Acute respiratory illness	<12 m: 83.6; 12-59 m: 39.4; 5-14 y: 11.4; 15-49 y: 6.9; ≥ 50 y: 5.2	
	[36]	Nicaraguan refugees in Costa Rica camp (1985)	Review of patient records	Acute respiratory infection		3.8 (1)
	[30]	Rwandan refugees in Zaire camps (1994)	Clinic-based surveillance	Acute respiratory infection		3.9 to 5.2 (≤ 3)
AURI	[36]	Nicaraguan refugees in Costa Rica camp (1985)	Review of patient records	Upper respiratory illness		1.6 (1)
	[51]	Earthquake-affected residents of Bam, Iran (2003)	Review of patient records	WHO case definition*		1.6 (1)
ALRI	[36]	Nicaraguan refugees in Costa Rica camp (1985)	Review of patient records	Lower respiratory illness		1.4 (2)
	[51]	Earthquake-affected residents of Bam, Iran (2003)	Review of patient records	WHO case definition		0.6 (3)
		
**Inpatient**						
ALRI	[38]	Cambodian refugees in Thai border camps (1987-1988)	Review of patient records	Pneumonia, croup or bronchiolitis	<15 y: 0.9 (1)	
	[39]	Cambodian refugees in Thai border camps (1989-1991)	Prospective case series	Empyema	<59 m: 0.009; 5-14 y: 0.005	

*Unspecified, but assumed to be based on Integrated Management of Childhood Illness guidelines.

Only one incidence report offered pre- and post-crisis comparisons: in a Nicaraguan population affected by a volcanic eruption [59], the post-eruption relative risks of consultation due to ARI as a whole, compared to before the disaster, were 3.6 to 6.0 overall depending on the site, 2.0 to 3.6 among infants <12 m, 2.6 to 6.1 among children 12 m-59 m, 6.0 to 7.4 among children 5-14 y, 5.2 to 10.0 among persons 15-49 y, and 7.7 to 10.0 among persons ≥ 50 y.

#### Proportional morbidity

Twenty-three articles reported on the proportional morbidity due to ARI (Table 2). However, only one described community-level morbidity, and 13 listed ARI as a whole without distinguishing ALRI.

**Table 2:** Reports of proportional morbidity due to ARI in crisis-affected populations, by setting (community, outpatient, inpatient).

**Disease**	**Ref**.	**Population (year)**	**Study design**	**Case definition, as reported**	**Percentage (rank if reported)**	
					**By age group**	**All ages**
		
**Community**						
AURI	[26]	Iraqi-Kurdish refugees in Iran camps (1991)	Community-based surveillance	Upper respiratory tract infection	<12 m: 23.3 (2); 12-59 m: 26.0 (2); ≥ 5 y: 15.2 (1)	17.5 (1)
ALRI	[26]	Iraqi-Kurdish refugees in Iran camps (1991)	Community-based surveillance	Lower respiratory tract infection	<12 m: 5.8 (3); 12-59 m: 3.8 (4); ≥ 5 y: 2.4 (5)	2.8 (9)
		
**Outpatient**						
ARI	[55]	Flood-affected residents of Bangladesh (1988)	Clinic-based surveillance	Respiratory tract infection	<59 m: 23.8 (2); 5-9 y: 17.8 (2); 10-14 y: 17.2 (2)	17.4 (2)
	[32]	Kosovar refugees in camps or with host families, Albania (1999)	Clinic-based surveillance	Acute respiratory infection	<59 m: 37; ≥ 5 y: 24 (1)	
	[34]	Kosovar refugees in Albania camp (1999)	Review of patient records	Upper and lower respiratory infection		22.0 (1)
	[33]	Kosovar refugees in camps or with host families, Albania (1999)	Clinic-based surveillance	Acute respiratory infection	<59 m: 40.2 (1); ≥ 5 y: 25.3 (1)	28.8 (1)
	[50]	Earthquake-affected residents of Nantou and Taichung counties, Taiwan (1999)	Clinic-based surveillance	Acute respiratory infection		50.1 (1)
	[56]	Flood-affected residents of Gaza province, Mozambique (2000)	Review of patient records	WHO case definition*		18 (2)
	[57]	Flood-affected residents of Orissa state, India (2001)	Clinic-based surveillance	Cold, cough, upper respiratory infection, pneumonia		26.9 (2)
	[44]	IDPs in camps or living with host population in Darfur state, Sudan (2004)	Clinic-based surveillance	Acute respiratory infection		18.7 (1)
	[58]	Tsunami-affected displaced persons in Banda Aceh, Indonesia (2004-2005)	Review of patient records	Upper and lower respiratory infection		39 (1)
	[54]	Earthquake-affected residents and displaced persons, northern Pakistan (2005)	Clinic-based surveillance	Acute respiratory infection	<59 m: 28 (1)**	22 (1)**
AURI	[34]	Kosovar refugees in Albania camp (1999)	Review of patient records	Upper respiratory infection		15.0 (2)
	[52]	Earthquake-affected residents of Bam, Iran (2003)	Clinic-based surveillance	Upper respiratory tract infection		
	[53]	Earthquake-affected people in Barakott, Pakistan (2005)	Review of patient records	Upper respiratory tract infection		14 (2)
ALRI	[37]	Afghan refugees in a camp in Pakistan (1986)	Review of patient records	Bronchitis, Pneumonia		9.2 (2)
	[29]	Burundian refugees in camps in Rwanda (1993-1994)	Clinic-based surveillance	Lower respiratory tract infection		6 (5)
	[34]	Kosovar refugees in Albania camp (1999)	Review of patient records	Lower respiratory infection		7.0 (5)
	[52]	Earthquake-affected residents of Bam, Iran (2003)	Clinic-based surveillance	Pneumonia		2.2 (8)
		
**Inpatient**						
ARI	[24]	Cambodian refugees in Khao-I-Dang holding centre, Thailand (1980)	Clinic-based surveillance	Pneumonia, bronchitis, upper respiratory infections	<20 y: 50.7 (1)	
	[41]	Sudanese refugees in Arua district, Uganda (1992-1994)	Review of patient records	Acute respiratory infection	<12 m: 26.9 (2); 12-59 m: 20.0 (2); 5-14 y: 21.0 (2); 15-49 y: 7.4 (5); ≥ 50 y: 8.5 (4)	13.6 (2)
	[48]	Urban population of Monrovia, Liberia (2005)	Review of patient records	Respiratory infection	≥15 y: 10 (1)	
AURI	[43]	Displaced persons in Gulu district, Uganda (1992-2002)	Review of patient records	Upper respiratory tract diseases		2.0 (10)
	[24]	Cambodian refugees in Khao-I-Dang holding centre, Thailand (1980)	Clinic-based surveillance	Upper respiratory infection	<20 y: 5.2 (7)	
ALRI	[42]	Displaced persons in Acholi region, Uganda (1992-1998)	Review of patient records	Pneumonia		5.2 (4)
	[43]	Displaced persons in Gulu district, Uganda (1992-2002)	Review of patient records	Pneumonia		6.4 (2)
	[38]	Cambodian refugees in Thai border camps (1987-1988)	Review of patient records	Pneumonia, croup or bronchiolitis	<15 y: 34.3 (1)	
	[24]	Cambodian refugees in Khao-I-Dang holding centre, Thailand (1980)	Clinic-based surveillance	Pneumonia	<20 y: 16.9 (2)	
	[39]	Cambodian refugees in Thai border camps (1989-1991)	Prospective case series	Empyema	<15 y: 3.5	
	[49]	Earthquake-affected residents of Kobe, Japan (1995)	Review of patient records	Pneumonia		15.9 (1)

*Unspecified, but assumed to be based on Integrated Management of Childhood Illness guidelines. **Data are averages for the first 12 months after the earthquake.

Where it was reported separately, ALRI was always within the top four causes of hospitalisation. In Kobe, Japan, during 15 days after a powerful earthquake, pneumonia was the first cause of hospitalisation among all ages combined. In northern Ugandan hospitals, pneumonia admissions rose two to three-fold concurrently with an increased intensity of armed conflict and displacement: during the same period, no such increase was noted in a control hospital in a non-conflict affected region of Uganda [42]. Considering all age groups, malaria, labour and delivery and tuberculosis in northern Uganda [42,43], and diarrhoea among Sudanese in northern Uganda [41], were more frequent than ALRI as hospitalisation causes.

ARI or AURI were consistently the first or second most frequent causes of outpatient consultation in all ages combined, and in children. Conditions more frequent than ARI or AURI included diarrhoea among Iraqi Kurdish refugees in Iran [26] and flood survivors in Orissa, India [57] and Bangladesh [55]; malaria after flooding in Mozambique [56]; and trauma after an earthquake in Pakistan [53] and forced displacement in Kosovo [34]. A further study [36] of Nicaraguan refugees in a Costa Rican camp (1985) showed that AURI and ALRI were the first (43.0%) and second (36.5%) most frequent infectious causes of consultation, respectively.

#### Other findings

A household survey from 1983 [35] measured the point prevalence of clinically diagnosed ARI contemporaneously among camp-living Guatemalan refugees and the neighbouring host population in Mexico. Prevalence among refugees peaked in children <12 m (34.4%) and 12-59 m (35.4%) old, and declined with age (5-14 y: 27.7%; 15-44: 13.3%; ≥ 45 y: 1.2%). These age-specific prevalences were, respectively, 1.6, 1.7, 2.2, 3.1 and 0.2 times those in the host population, suggesting the greatest excess risk occurred in older children and adults.

No data on the aetiology of ARI in crises were found. One study [40] among Vietnamese refugee children under 5 y in a Hong Kong camp showed that 61% of those diagnosed with measles during a 1991-1992 outbreak went on to develop pneumonia, but no comparison with the host population is available.

### ARI-attributable mortality

#### Cause-attributable mortality rate

Only three reports provided data on the ARI-attributable population mortality rate. Among Bhutanese refugees in Nepal in 1992-1993, 0.29 deaths per 10 000 person-days were due to ALRI, defined as fever, cough and >50 breaths per minute, making ALRI the leading cause of death over the 6 months analysis period [28]. In the same population and over a partly overlapping period, ARI-attributable mortality rates were 0.5 per 10 000 person-days among all ages, 1.6 among children under 5 y and 0.3 in older persons [27]. Among Burundian refugees in Rwanda (1993), the mortality rate attributable to ALRI was 0.2 per 10 000 person-days [29].

Proportional mortality

Sixteen reports provided data on the proportion of deaths due to ARI (Table 3), though eleven only provided health-facility-based data. ARI as a whole or ALRI were among the top three causes of death in all but one study, irrespective of age group. Considering community-based studies only, proportional mortality was extremely high among Bhutanese in Nepal (≥ 40%). High percentages among children older than 5 y were noted in Bangladesh (16-17%) and among Rwandans in Zaire (33%). The only cause of death more frequent than ARI was diarrhoea among flood survivors in Bangladesh [55], Burundians in Rwanda [29] (epidemic dysentery) and Rwandan children in Zaire [31] (epidemic cholera).

**Table 3:** Reports of proportional mortality due to ARI in crisis-affected populations, by setting (community, inpatient).

**Disease**	**Ref**.	**Population (year)**	**Study design**	**Case definition, as reported**	**Percentage (rank if reported)**	
					**By age group**	**All ages**
		
**Community**						
ARI	[55]	Flood-affected residents of Bangladesh (1988)	Community-based surveillance	Respiratory tract infection	<12 m: 4.7 (3); 12-59 m: 16.2 (2); 5-9 y: 16.7 (2); 10-14 y: 0 (n/a); 15-44 y: 8.3 (5); ≥ 45 y: 18.0 (2)	13.0 (2)
	[27]	Bhutanese refugees in Nepal camps (1992)	Community-based surveillance with verbal autopsies	Acute respiratory infection		55 (1)
ALRI	[28]	Bhutanese refugees in Nepal camps (1992)	Community-based surveillance with verbal autopsies	Fever, cough and rapid breathing at death without evidence of measles		40 (1)
	[29]	Burundian refugees in Rwanda camps (1993)	Community- and graveyard-based surveillance	Lower respiratory tract infection		6 (3)
	[31]	Unaccompanied Rwandan refugee children in Zaire (1994)	Orphanage-based surveillance	Pneumonia	<15 y: 33 (2)	
		
**Inpatient**						
ARI	[41]	Sudanese refugees in Uganda camps (1992-1994)	Review of patient records	Acute respiratory infection	<12 m: 25.0 (2); 12-59 m: 32.0 (2); 5-14 y: 30.8 (2); 15-49 y: 16.0 (2); ≥ 50 y: 0 (n/a)	23.9 (2)
	[32]	Kosovar refugees in Albania (1999)	Clinic-based surveillance	Acute respiratory infection	<59 m: 36 (1)	13 (2)
	[47]	Urban population of Herat, Afghanistan (2002-2003)	Review of patient records	Acute respiratory infection	Paediatric: 30.5 (1)	
	[46]	Urban population of Kabul, Afghanistan (2002-2003)	Review of patient records	Acute respiratory infection	1 m-12 y: 22 (2)	
	[71]	Earthquake-affected residents and displaced persons, northern Pakistan (2005)	Clinic-based surveillance	Acute respiratory infection		26 (1)
	[48]	Urban population of Monrovia, Liberia (2005)	Review of patient records	Respiratory infection	1-59 m: 31 (1); 5-14 y: 5 (7)	
ALRI	[24]	Cambodian refugees in Khao-I-Dang holding centre, Thailand (1980)	Clinic-based surveillance	Pneumonia	<20 y: 11.6 (3)	
	[25]	Somali refugees in Ethiopia camps (1989)	Clinic-based surveillance	Acute lower respiratory infection, pneumonia	<59 m: 34 (2)	
	[49]	Earthquake-affected residents of Kobe, Japan (1995)	Review of patient records	Pneumonia		22.9 (2)
	[42]	Displaced persons in Acholi region, Uganda (1992-1998)	Review of patient records	Pneumonia		7.4 (3)
	[43]	Displaced persons in Gulu district, Uganda (1992-2002)	Review of patient records	Pneumonia		9.1 (3)

Considering inpatient data, percentages >20% were the rule among all populations but all-age patients in northern Uganda. Causes of death more frequent than ARI included malnutrition among Ugandan IDPs [42,43] and Cambodians in Thailand [24]; malaria among Ugandan IDPs [42,43], Burundians in Rwanda [29], and older children in Liberia [48]; diarrhoea among Sudanese in Uganda [41] and Somalis in Ethiopia [25]; measles among Cambodians in Thailand [24]; sepsis or septicaemia among children in Liberia [48] and Afghanistan [46]; heart disease among adult Kosovars in Albania [32]; cancer among earthquake survivors in Japan [49]; and surgical complications, tetanus and trauma among older children in Liberia in a referral hospital [48].

Only one study contained a comparison with a non-crisis affected control population. In a hospital in war-affected northern Uganda, the proportion of mortality due to pneumonia was similar (7.4%) to that in twenty other non-war affected Ugandan hospitals (7.9%), though in the same hospital many pneumonia-attributable deaths may have been classified as malnutrition, which was the first cause of inpatient deaths with 13.1% [42].

#### Case-fatality

We found seven reports of ARI CFR, all from inpatient settings (Table 4). CFR was consistently above 9% except for one study [39] that considered only empyema cases. The highest CFRs were noted among Sudanese refugees in Uganda (>30% in all but the youngest age group). In a paediatric ward in Guinea Bissau, pre-war CFR due to all diseases, and adjusted for various confounders, was higher than during the period of active fighting, possibly due to increased equity of care and availability of free drugs during the ensuing humanitarian response; the pneumonia CFR showed a similar trend (17.2% before the war, 13.1% during) [45]. This is unlikely to be due to self-selection of milder cases, as bed occupancy was actually higher during the war period.

**Table 4:** Reports of the case-fatality ratio of ARI in crisis-affected populations (inpatient only).

**Disease**	**Ref**.	**Population (year)**	**Study design**	**Case definition, as reported**	**Case-fatality ratio (%)**	
					**By age group**	**All ages**
		
ARI	[41]	Sudanese refugees in Uganda camps (1992-1994)	Review of patient records	Acute respiratory infection	<12 m: 9.5; 12-59 m: 33.8; 5-14 y: 41.7; 15-49 y: 30.9	31.3
	[46]	Urban population of Kabul, Afghanistan (2002-2003)	Review of patient records	Acute respiratory infection	1 m-12 y: 16	
	[48]	Urban population of Monrovia, Liberia (2005)	Review of patient records	Respiratory infection	1 m-14 y: 12; ≥ 15 y: 10	
ALRI	[39]	Cambodian refugees in Thai border camps (1989-1991)	Prospective case series	Empyema	<15 y: 1.0	
	[49]	Earthquake-affected residents of Kobe, Japan (1995)	Review of patient records	Pneumonia		12.9
	[45]	Urban population of Bissau, Guinea Bissau (1998-1999)	Clinic-based surveillance	Pneumonia	<15 y: 13.1	
	[43]	Displaced persons in Gulu district, Uganda (1992-2002)	Review of patient records	Pneumonia		11.2

## Discussion

This systematic review provides evidence that ARI is a leading cause of morbidity and mortality in crises. This finding appears consistent across various types of crisis, including natural disasters, and phases of the emergency. As expected, the greatest burden is in children <12 m. However, we also found that ARI is usually among the top two causes of morbidity and mortality among older age groups.

### Comparisons with non-crisis settings

Only 4 of 36 studies provided a direct comparison with non-crisis settings. These suggest a much increased incidence and prevalence of ARI. Both studies comparing burden by age group suggested that the greatest excess risk occurred in children 5-14 y and adults. One study showed that pneumonia admissions rose during wartime whilst in neighbouring peaceful settings they did not, although the proportional mortality was similar. One study suggested a slightly lower CFR during the crisis, compared to the pre-crisis period.

Other comparisons with non-crisis settings are arduous, and can only be made indirectly by comparing findings with corresponding global non-crisis estimates. None of the studies reported on community incidence among children under 5 y, estimated globally at around 5 ALRI episodes per 1000 child-weeks [8]. While proportional morbidity data establish the importance of ARI as a major cause of both consultations and hospitalisations in an absolute sense, we do not know of comparable global estimates of the health-facility based burden of ARI disease.

ARI-attributable mortality rates were only available from two crises: compared to the expected mortality rates considering country-specific crude death rates [60] and regional burden of ARI disease estimates [61] over the same time period, these observed rates were roughly 10-17 times higher in Nepal and four times higher in Burundi.

In our review, the CFR of ALRIs (which may not be restricted to severe pneumonia) appeared considerably higher than in non-crisis settings, where the CFR of severe pneumonia was <14% (median 10%) in studies reviewed by Rudan [8], <6% in Bangladesh [62],<7% in Fiji [63], and 16% in Uganda [64].

Worldwide, the proportion of deaths attributable to ARI among children under 5 y is estimated at between 17% and 23% depending on the source, and on whether ALRI other than pneumonia as well as ALRI's contribution to neonatal deaths are also included [11,65]. This proportion was somewhat higher (20-35%) in most studies we reviewed from crisis settings, though ARI was often second to diarrhoea. Similarly, considering all age groups combined, we found considerably higher proportional inpatient mortality from ALRI (9-26% among all ages, 25-36% among children under 5 y) in nearly all studies reviewed than the 7% and 17% estimated worldwide on a population level, respectively [14]. However, comparisons of inpatient and population-based data should be made with caution, as the probability of inpatient admission is unlikely to be the same for every cause of mortality. On balance, given that in the acute phase of crises all-cause mortality is frequently double or more the pre-crisis baseline [66], our finding that the ARI proportional mortality in crises is similar or greater than in stable settings suggests that in crises the risk of dying from ARI increases at least as much, and perhaps more than that of other common diseases.

### Limitations of this review

The most important limitation of this review is that nearly all studies reviewed did not describe how ARI diagnosis was made, while many probably did not rely on a standard case definition. While most studies classified tuberculosis separately, other respiratory illnesses such as asthma may have been included among ARI diagnoses (while we excluded studies in which respiratory illness was not stratified into infectious and non-infectious, particularly in infants and neonates this distinction is difficult to make because ARI sometimes presents without fever, and children with acute asthma are often febrile on presentation.). In stable malaria transmission settings, the ARI-malaria symptom overlap is well described [67]. Febrile illness is often treated presumptively as malaria [68], particularly in resource-constrained relief settings, probably leading to under-reporting of the true ARI burden. Furthermore, all studies we reviewed reported only a single cause of disease or death, whereas in fact many childhood deaths are due to multiple pathologies. Death from ARI is often associated with malnutrition, measles or HIV/AIDS.

Systematic differences in crisis and non-crisis populations' age distributions hamper both direct and indirect comparisons. If crisis-affected populations were on average younger, this lack of age standardisation might result in over-estimation of the relative ARI risk in the crisis affected settings. Similarly, crisis settings might already feature a higher baseline disease burden, biasing the indirect comparison towards an overestimation of the crisis-attributable relative risk.

Different selection biases may affect the representativeness of our findings. Firstly, the populations and crisis settings covered by the reports we reviewed may not be representative of worldwide patterns. Populations in the immediate aftermath of acute emergencies, especially war-related, are rarely the subject of in-depth epidemiological investigations, despite the fact that excess (and preventable) morbidity and mortality are highest in this phase. This review included mainly reports from refugees in camps, who in fact comprise only a minority of crisis-affected people in any given year; evidence on IDPs and non-displaced populations living in insecure settings is scarce: it is plausible that ARI burden in these populations would be higher than in camps, due to insufficient humanitarian access, lower vaccination coverage, and extended periods of nutritional crisis. In general, the effect of humanitarian assistance may have to varying degrees confounded the true impact of crises on ARI burden, so that our findings mainly represent burden in settings benefiting from some humanitarian relief.

Secondly, data on outpatient and inpatient proportional morbidity and mortality may not be representative of population patterns, due to differences in health care utilisation by type of illness. These data do, however, provide some information on the contribution of ARI to the patient caseload that relief agencies should expect in a variety of crisis scenarios.

A major finding of this review is that the types of ARI data collected in crises are mostly not very useful to assess the relative and absolute burden, and draw comparisons with non-crisis settings. For example, only half (18/36) of studies reviewed provided information on ALRI specifically, and, of these, very few (4/18) age-stratified data at least into children under 5 y and older persons.

## Conclusions

Our review suggests that the burden of ARI, already very large in stable settings, increases considerably in crises. This pattern appears consistent across different types of crisis, including natural disasters. In the latter, the risk of infectious disease epidemics is usually considered to be low [69], but this may lead to neglect of common conditions such as ARI.

ARIs are less noticeable than epidemic-prone diseases in crises, and any abnormal increases are difficult to detect against a background of consultations for fever and rapidly evolving health facility utilisation rates. This reflects in part a perception by humanitarian workers, mostly based on models of refugee camp health care developed in the 1980s, that infectious disease threats in crises are essentially from easily recognisable and dramatic epidemics of cholera, measles or meningitis Large epidemics of some ARI pathogens may nonetheless occur, and in general ARI pathogens should be considered epidemic-prone in crises, though diagnostics to confirm these epidemics may not be available. The true impact of ARIs is a function of both incidence and case-fatality. There are no acceptable targets for ARI CFR, unlike for cholera or severe malnutrition, making it difficult to monitor the quality of case management on the basis of accepted standards. Further contributing to ARIs' neglect in crisis settings, surveillance systems set up in emergencies generally focus on early detection of visible epidemic-prone diseases. While data on ARIs are often collected, in our experience they are seldom used to inform action.

Our findings of high burden in older children and adults are highly relevant for vaccination strategies, particularly with pneumococcal, Hib, measles and pertussis vaccines. Older children are rarely included in target age groups for these vaccines, but our findings suggest that they perhaps should be, at least in crisis situations.

As advocated for stable settings [70], better characterisation of the epidemiology and aetiology of ARI and particularly pneumonia in crisis-affected settings is critical to rationalise disease priorities, gauge the potential impact of improved diagnostics and treatment, optimise treatment algorithms, and make the best use of available and new vaccines against Hib, pneumococcus, measles and pertussis. Future studies should focus on ALRI; implement clear and standardised case definitions (e.g. clinical versus radiological pneumonia); age-stratify data (with finer strata among children below 5 y so as to better characterise age distribution and optimise vaccine target groups accordingly); and describe the morbidity and mortality burden at the population level rather than based on health-facility data alone. The latter will require focussed community surveillance studies, accompanied by verbal autopsies. Since certain pathogens and serotypes responsible for ARI may be particularly favoured by risk factors such as overcrowding or acute malnutrition, appropriately resourced aetiological studies should also be implemented in a selection of sites.

Addressing ARI in crises is key to achieving global child survival targets and Millennium Development Goals. Accordingly, initiatives such as the WHO and UNICEF-led Global Action Plan for Pneumonia prevention and control (GAPP) need to extend their reach to humanitarian relief settings. Agencies working in crisis settings should invest greater resources in ARI prevention and control, and explicitly consider ARI and pneumonia a top priority across crisis phases and scenarios. Similarly, ARI prevention and treatment should become part of the standard package of minimum public health interventions in crises.

## Competing interests

The authors declare that they have no competing interests.

## Authors' contributions

AB and FC designed the review. AB carried out the review. FC wrote the paper. KM, KLoB, SAQ and MG interpreted findings and made contributions to the paper. All authors read and approved the final manuscript.

## Supplementary Material

Additional file 1 **The burden of acute respiratory infections in crisis-affected populations: a systematic review**. Box 1. Subject heading and keywords (number of corresponding abstracts) for the MeSH search. Box 2. Subject heading and keywords (number of corresponding abstracts) for the armed conflict-specific search: example of Afghanistan. Box 3. List of 37 war-affected countries included in the armed conflict-specific search (number of corresponding abstracts). Box 4. Subject heading and keywords (number of corresponding abstracts) for the disaster-specific search. Click here for file
