# Supplementary material for: The burden of acute respiratory infections in crisis-affected populations: a systematic review
Source: Confl Health. 2010 Feb 11;4:3. doi: 10.1186/1752-1505-4-3 (PMC2829474; doi:10.1186/1752-1505-4-3)
Supplement: Additional file 1 — The burden of acute respiratory infections in crisis-affected populations: a systematic review. Box 1. Subject heading and keywords (number of corresponding abstracts) for the MeSH search. Box 2. Subject heading and keywords (number of corresponding abstracts) for the armed conflict-specific search: example of Afghanistan. Box 3. List of 37 war-affected countries included in the armed conflict-specific search (number of corresponding abstracts). Box 4. Subject heading and keywords (number of corresponding abstracts) for the disaster-specific search. [file 1752-1505-4-3-S1.DOC]

**The burden of acute respiratory infections in crisis-affected populations: a systematic review**

**Additional File**

Box 1. Subject heading and keywords (number of corresponding abstracts) for the MeSH search.

| 1. exp Respiratory Tract Infections/ (351523) 2. exp Pneumonia/ or exp Pneumonia, Bacterial/ (157863) 3. pneumonia.mp. (189496) 4. bacterial pneumonia.mp. (8334) 5. acute lower respiratory tract infection.mp. (318) 6. acute respiratory tract infection.mp. (455) 7. ARI.mp. (2615) 8. ALRI.mp. (374) 9. exp Influenza, Human/ (37866) 10. influenza.mp. (97657) 11. respiratory.mp. (733562) 12. 6 or 11 or 3 or 7 or 9 or 2 or 8 or 1 or 4 or 10 or 5 (1104505) 13. exp Epidemiology/ (951274) 14. exp Mortality/ (504040) 15. exp Morbidity/ (359223) 16. exp Incidence/ (271134) 17. exp Prevalence/ (287249) 18. (case fatality or case fatality rate).mp. (8348) 19. (infant mortality or child mortality).mp. (33430) 20. cause of death.mp. (91798) 21. excess mortality.mp. (5347) 22. CFR.mp. (3115) 23. disease burden.mp. (4488) 24. health.mp. (2348785) 25. case$.mp. (5096058) 26. episode$.mp. (217239) 27. incidence.mp. (893182) 28. prevalence.mp. (632060) 29. illness.mp. (355948) 30. attack rate.mp. (3545) 31. outbreak.mp. (64537) 32. epidemic.mp. (97292) 33. morbidity.mp. (363447) 34. epidemiology.mp. (327815) 35. (public health or public health impact).mp. (244868) 36. infection.mp. (1666671) 37. mortality.mp. (791987) 38. mortality rate.mp. (82510) 39. 13 or 14 or 15 or 16 or 17 or 18 or 19 or 20 or 21 or 22 or 23 or 24 or 25 or 26 or 27 or 28 or 29 or 30 or 31 or 32 or 33 or 34 or 35 or 36 or 37 or 38 (10078915) 40. exp Refugees/ (8998) 41. exp War/ (39715) 42. exp Disasters/ (58917) 43. exp Earthquakes/ (753) 44. (internally displaced population or internally displaced person$ or displaced population).mp. (262) 45. refugee$.mp. (11602) 46. (crisis-affected population$ or crisis affected population$).mp. (4) 47. armed conflict.mp. (565) 48. (complex emergencies or complex emergency).mp. (272) 49. forced displacement.mp. (68) 50. forced migration.mp. (172) 51. natural disaster$.mp. (4813) 52. humanitarian.mp. (3370) 53. refugee camp.mp. (554) 54. (conflict and health).mp. (15052) 55. war.mp. (56734) 56. 40 or 41 or 42 or 43 or 44 or 45 or 46 or 47 or 48 or 49 or 50 or 51 or 52 or 53 or 54 or 55 (148400) 57. 39 and 56 and 12 (3846) 58. Limit 57 to yr="1980 -Current" (3727) 59. Remove duplicates from 58 (3268) |
| --- |

Box 2. Subject heading and keywords (number of corresponding abstracts) for the armed conflict-specific search: example of Afghanistan.

| 1. war.mp. (56734) 2. disease.mp. (4954724) 3. respiratory.mp. (733562) 4. 3 or 2 (5474240) 5. 4 and 1 (7355) 6. Afghanistan.mp. (5267) 7. 6 and 5 (233) 8. Remove duplicates from 7 (168) |
| --- |

Box 3. List of 37 war-affected countries included in the armed conflict-specific search (number of corresponding abstracts).

| Afghanistan (168)  Algeria (7)  Angola (31)  Azerbaijan (8)  Bosnia and Herzegovina (159)  Burundi (23)  Chad (8)  Congo (51)  Democratic Republic of Congo (Zaire)(45)  Eritrea (7)  Ethiopia (45)  Georgia (8)  India (74)  Indonesia (20)  Iraq (311)  Lebanon (70)  Liberia (15)  Libya (3)  Myanmar (21)  Nepal (14)  Nicaragua (10)  Pakistan (31)  Peru (12)  Sudan (79)  Tajikistan (0)  Turkey (40)  Uganda (42)  USA (n/a)  Yemen (8)  Yugoslavia (Serbia) (123)  Philippines (20)  Russia (Soviet Union) (163)  Rwanda (46)  Sierra Leone (17)  Somalia (46)  South Yemen (see Yemen)  Sri Lanka (Ceylon) (29)  Total = 1754 abstracts. |
| --- |

Box 4. Subject heading and keywords (number of corresponding abstracts) for the disaster-specific search.

| 1. acute lower respiratory tract infection.mp. (318) 2. respiratory.mp. (733562) 3. health.mp. (2348785) 4. illness.mp. (355948) 5. infection.mp. (1666671) 6. tsunami.mp. (1723) 7. earthquake.mp. (4184) 8. flood$.mp. (45394) 9. drought.mp. (51625) 10. famine.mp. (3713) 11. volcano.mp. (2422) 12. volcanic.mp. (7794) 13. 11 or 12 (9206) 14. disease.mp. (4954724) 15. 4 or 3 or 14 or 5 (7945310) 16. 1 or 2 (733562) 17. 6 and 16 and 15 (46) 18. 7 and 16 and 15 (84) 19. 8 and 16 and 15 (190) 20. 16 and 9 and 15 (53) 21. 16 and 10 and 15 (15) 22. 16 and 13 and 15 (105) 23. Remove duplicates from 17 (39) 24. Remove duplicates from 18 (70) 25. Remove duplicates from 19 (138) 26. Remove duplicates from 20 (37) 27. Remove duplicates from 21 (11) 28. Remove duplicates from 22 (75) |
| --- |
